# Reconsidering the need for gain-of-function research on enhanced potential pandemic pathogens in the post-COVID-19 era

**DOI:** 10.3389/fbioe.2022.966586

**Published:** 2022-08-26

**Authors:** Nariyoshi Shinomiya, Jusaku Minari, Go Yoshizawa, Malcolm Dando, Lijun Shang

**Affiliations:** ^1^ National Defense Medical College, Saitama, Japan; ^2^ Uehiro Research Division for iPS Cell Ethics, Center for iPS Cell Research and Application (CiRA), Kyoto University, Kyoto, Japan; ^3^ Innovation System Research Center, Kwansei Gakuin University, Hyogo, Japan; ^4^ Section of Peace Studies and International Development, University of Bradford, Bradford, United Kingdom; ^5^ School of Human Sciences, London Metropolitan University, London, United Kingdom; ^6^ Biological Security Research Center, London Metropolitan University, London, United Kingdom

**Keywords:** potentially pandemic pathogens, gain-of-function research, COVID-19, governance, risk/benefit

## Abstract

The dual-use risk of infectious disease research using enhanced potential pandemic pathogens (ePPP), particularly gain-of-function (GOF) research, has been debated since 2011. As of now, research is supported on the condition that the research plan is reviewed and the actual experiment is supervised. However, the kinds of research conducted and what benefits they have brought to our society have not been adequately verified. Nevertheless, due to the COVID-19 pandemic that began at the end of 2019 and caused numerous deaths and wide economic disruption, the importance of infectious disease control from an international perspective has been recognized. Although complete control of the pandemic is still far off, positive signs include generating epidemiological trends based on genome analysis, therapeutic drug and vaccine development, clinical patient management, and public health policy interventions. In this context, the time has come to reconsider the true significance of GOF research on ePPP and the state of research governance in the post-COVID-19 era. In particular, the risks of such research are clearer than before, whereas its benefits seem less apparent. In this paper, we summarize the history of discussions on such GOF research, its significance in the light of the current COVID-19 pandemic, and the direction we shall take in the future.

## Introduction

COVID-19, which began at the end of 2019, has quickly spread worldwide, plunging many people into the depths of sickness and causing countless deaths. Urban lockdowns have devastated economies, and suicides are on the rise due to economic deprivation and an increase in depression (Sher, 2020). Face-to-face interactions between people were cut off, and events such as conferences and gatherings for business were canceled at every turn. Although more and more opportunities exist to connect online as a means of contact, there is also a problem of what is called the digital divide, where people are left out of these opportunities. Vaccination, which started at the end of 2020, has been a light at the end of the tunnel; however, there is still a long way to escape from the tunnel.

COVID-19 is a respiratory disease caused by the SARS-CoV-2 virus. As the disease progresses, it can turn into a systemic disease (known as long COVID), resulting in pneumonia and vascular disorders. For some time now, humanity has been drastically changing the global environment through overexploitation, which has brought dramatic changes in the nature of the interaction between plants, animals, and humans. The concept of “one health” (UN Enviroment Programme, 2021) has been introduced in relation to the control of emerging and reemerging infectious diseases because such global environmental changes have altered ecosystems, which have brought about changes in infectious disease outbreaks. The origin of the SARS-CoV-2 virus, the cause of COVID-19, is currently unknown. Whether it arose naturally or through human error, the fact remains that we must strengthen our public health infrastructure to deal with the current pandemic, and we must also be prepared for any new emerging infectious diseases that may arise in the future.

In this context, we are at a crossroads at which researchers must discuss with society how infectious disease research should be conducted as we enter a new post-COVID-19 era. In particular, with regard to a type of infectious disease research called gain-of-function (GOF) research on enhanced potential pandemic pathogens (ePPP), it is necessary to explain it—and its benefits, if any—more thoroughly to the society as a whole (Selgelid, 2016; Kozlov, 2022).

Especially because of the current COVID-19 pandemic, we must seriously consider what kind of infectious disease research is meaningful and what kind of research poses major risks and provides little benefit. This article focuses on this important issue and calls for a rethinking of how GOF research is conducted. Notably, several published papers have examined the possibility that GOF research gave rise to SARS-CoV-2 in China, but this paper is not intended to pursue this point in depth.

## The origin of GOF research

The term “gain-of-function” means “to enhance a function by genetic manipulation” or “to add a new function” and applies to much research involving genetic recombination and genetic manipulation. However, in the study of pathogens in infectious diseases, especially in influenza research, mutations in viral genes may cause changes in pathogenicity, infectivity, transmissibility, host range, etc., and thus alter the characteristics of the disease. Therefore, research is being conducted to deliberately introduce mutations and explore them. On the one hand, the purpose of such research is to detect mutant strains as soon as possible and use them for surveillance, epidemic forecasting, and vaccine development. It is also used to improve our understanding of the mechanisms by which pathogens function. On the other hand, those studies have been the subject of debate on the ambiguity of the use of research results (the so-called dual-use problem) because of the possible diversion of research results to biological weaponization, as well as the escape of the pathogens under study from containment, causing an outbreak that can lead to a pandemic.

In addition, a number of recent advances in life science technologies, such as recombinant DNA technology, sequencing technology, and DNA synthesis technology, have led to the emergence of reverse genetics and synthetic biology, which have been the driving forces behind the rapid development of GOF research with ePPPs since 2000. The terrorist attacks and the anthrax mailings in the United States in 2001 raised the importance of biosecurity and biopreparedness as well as infectious disease crisis management in public health and provided the impetus to broaden the existing legislative controls governing the transfer of dangerous pathogens to a program tasked with regulating their possession and use (i.e., the Select Agent program). In particular, the research that led to the development of a strain of mousepox that defeated both vaccines and preexisting immunity (Jackson et al., 2001) suggested that the same technology could be used against the human smallpox virus, and a great deal of debate ensued about the pros and cons of conducting this type of research. At the same time, a number of studies raised the issue of dual-use, such as the complete chemical synthesis of poliovirus (Cello et al., 2002) and the cloning of the SPICE gene, one of the virulence factors of the smallpox virus (Rosengard et al., 2002), which were the subject of discussion in *Biotechnology Research in an Age of Terrorism* (also known as the Fink report) (Committee on Research Standards and Practices to Prevent the Destructive Application of Biotechnology, 2004). The Fink report was the first report that shook the nature of pathogen research to its very core. Since then, the Fink report’s “Seven classes of experiments” (Table 1) have been used as criteria for determining the existence of dual-use research of concern (DURC) in pathogen research. With the report of the artificial reconstruction of the 1918 Spanish flu virus (Tumpey et al., 2005), a new DURC category was added to the list, which includes not only the manipulation of pathogenicity through genetic modification but also the creation of pathogens by synthesizing genomes.

**TABLE 1:** “Seven classes of experiments” identified in the Fink report.

Experiments that:	
1	Would demonstrate how to render a vaccine ineffective
2	Would confer resistance to therapeutically useful antibiotics or antiviral agents
3	Would enhance the virulence of a pathogen or render a nonpathogen virulent
4	Would increase the transmissibility of a pathogen
5	Would alter the host range of a pathogen
6	Would enable the evasion of diagnostic/detection modalities
7	Would enable the weaponization of a biological agent or toxin

Infectious diseases are caused by a combination of three factors: the nature of the pathogen (infectivity), the environmental factors that transmit the pathogen (transmission pathway), and the susceptibility of the host (host susceptibility). In this context, GOF research has developed as a type of research that manipulates the properties of pathogens, especially their infectivity.

Genetic engineering in infectious disease research to change infectious agents has evolved uniquely due to the combination of both advances in gene synthesis technology and methods of reconstituting viruses using reverse genetics techniques, which led to the use of the term GOF. Historically, in addition to influenza viruses, which have been the cause of many pandemics in the past, severe acute respiratory syndrome (SARS) and the Middle East respiratory syndrome (MERS) can also cause pandemics, making GOF research on these three infections a particular focus of biosafety and biosecurity attention. What we would like to make clear is the definition of GOF research discussed in this paper. While some other publications may have interpreted it differently, we define it as follows:

*GOF research in this paper refers to the study of ePPPs, which are potential pandemic pathogens (PPPs) that have been created by enhancing their transmissibility or virulence. The starting point for this enhancement need not already be a PPP, so long as the end result is. In most cases, viruses causing influenza, SARS, and MERS have been used for experiments to modify the genomes of pathogens to create new strains of those pathogens. Creating new strains of existing pathogens for which genomic information is known by synthetic biology or reverse genetics is also possible. Still, the risks of these methods need to be discussed from a different perspective from that of GOF research.*

## Discussions about GOF research and the framework for research support

First, we would like to review the debate over GOF research from 2011 to 2013. In November 2011, Dr. Yoshihiro Kawaoka of the University of Wisconsin-Madison School of Veterinary Medicine submitted a paper to *Nature*. Dr. Ron Fouchier of the Erasmus Medical Center in the Netherlands submitted a paper to *Scienc*e (Enserink, 2011). These papers detailed the genetic modification of avian influenza viruses to alter the host ranges from birds to mammals. The editors of both journals requested the National Science Advisory Board for Biosecurity (NSABB) (Table 2), a United States government-wide advisory committee affiliated with the National Institutes of Health (NIH) that has a broader charter and mandate, to conduct a biosecurity review. At that time, there was no precedent in the scientific community that research results derived from a research project approved by a public grant could be withheld from publication after the submission due to the risk of dual-use.

**TABLE 2:** Composition of NSABB.

Voting members
25 members with a broad range of expertise in molecular biology, microbiology, infectious diseases, biosafety, public health, veterinary medicine, plant health, national security, biodefense, law enforcement, scientific publishing, and related fields
Nonvoting ex officio members
15 federal agencies and departments

In January 2012, researchers working on influenza announced a voluntary moratorium on animal experiments related to GOF research, which was initially proposed to have a duration of 60 days, in an attempt to gain public understanding (Fouchier et al., 2012). In contrast, in light of the current situation where a short-term solution was unlikely, the importance of continuing research was stressed by the researchers involved in GOF research, who stated that “Flu transmission work is urgent” even during the moratorium (Kawaoka, 2012). In February of the same year, the World Health Organization (WHO) convened a meeting of H5N1 influenza researchers and decided not to limit the resolution of this issue to the 60-day moratorium period that had been initially set (Schnirring, 2012). The debate on the merits of publishing these two influenza papers was finally resolved through direct hearings with the authors at the NSABB in March, resulting in their acceptance and publication (Collins, 2012). The recommendation of the NSABB was that the revised manuscript submitted by Dr. Yoshihiro Kawaoka be communicated in full, whereas the data, methods, and conclusions presented in the revised manuscript submitted by Dr. Ron Fouchier be communicated after appropriate scientific review and revision. After stating the advantages of both influenza studies in that they were unlikely to be immediately misused and may contribute to surveillance and biopreparedness, it was added that there is a need for management and oversight of research in this area and that efforts should be made to promote public understanding of the dual-use issue (National Science Advisory Board for Biosecurity, 2012).

However, the recommendation for both papers by the NSABB and the decision to publish them in journals did not calm the debate on the merits of GOF research. Since then, the debate has continued to divide the world’s virus researchers, and many symposia and workshops have been held in relation to this issue. Under these circumstances, the voluntary moratorium, first limited to 60 days, was eventually continued for 1 year.

In December 2012, a workshop entitled “Gain-of-Function (GOF) Research on Highly Pathogenic Avian Influenza (HPAI) H5N1 Viruses: An International Consultative Workshop” was held at the NIH in Bethesda, United States. Leading researchers from around the world whose work was relevant to the discussion of GOF research were invited. The goal was to hear the views of various stakeholders through discussions from an interdisciplinary and international perspective, especially to understand what approaches other governments and funding agencies were taking to advance GOF research on highly pathogenic avian influenza H5N1 and to propose a new funding framework for the United States Department of Health and Human Services (HHS).

Perhaps influenced by the atmosphere of discussion at the workshop, in which the GOF research was seen in the most positive light, in January of 2013, influenza researchers issued a statement that the purpose of the moratorium had been achieved, it would be lifted, and animal experiments in influenza GOF research would begin (Fouchier et al., 2013a; Fouchier et al., 2013b). The researchers stated that “we declare an end to the voluntary moratorium on avian-flu transmission studies” and that “researchers who have approval from their governments and institutions to conduct this research safely, under appropriate biosafety and biosecurity conditions, have a public health responsibility to resume this important work.” They concluded that “because the risk exists in nature that an H5N1 virus capable of transmission in mammals may emerge, the benefits of this work outweigh the risks”. However, this hasty conclusion left many scientists with misgivings (Butler, 2013).

In support of lifting the moratorium, the results of the December 2012 NIH workshop were published in the “Policy Forum” of *Science* in March 2013 (Patterson et al., 2013). The conclusion was that if the seven criteria listed in Table 3 were met, the government could provide public research funding; thus, these criteria were used from then on.

**TABLE 3:** Criteria for guiding HHS funding decisions for the creation of H5N1 GOF research proposals (Patterson et al., 2013).

•	Such a virus could be produced through a natural evolutionary process
•	The research addresses a scientific question with high significance to public health
•	There are no feasible alternative methods to address the same scientific question in a manner that poses less risk than does the proposed approach
•	Biosafety risks to laboratory workers and the public can be sufficiently mitigated and managed
•	Biosecurity risks can be sufficiently mitigated and managed
•	The research information is anticipated to be broadly shared to realize its potential benefits to global health
•	The research will be supported through funding mechanisms that facilitate appropriate oversight of the conduct and communication of the research

However, this was the conclusion of NIH officials and not necessarily the unanimous opinion of all researchers. Therefore, it was impossible to convince the researchers who were concerned about the current state of GOF research. HIV researcher Simon Wain-Hobson of the Pasteur Institute immediately published an article in *Nature* titled “H5N1 viral-engineering dangers will not go away” (Wain-Hobson, 2013) arguing that “governments, funders, and regulators must urgently address the risks posed by GOF research.” In the first place, he argued that the withdrawal of the moratorium (ending after 1 year) was initiated by the relevant influenza researchers, and the opinions of funders, governments, and international organizations were not reflected in the decision. He also raised concerns about the impact on infectious disease research as a whole, depending on the impression of GOF research that science policymakers have. The questions he posed were as follows:

1. Is the virological basis for the study solid?
2. Who makes the rules, and who oversees them?
3. If the research produces a highly pathogenic and transmissible virus, how will the research be handled?
4. If the pathogen leaks and an outbreak occurs, who will be responsible and compensated?
5. Is it really appropriate to modify the microorganism to make it more dangerous? Are funders and regulators negligent in their duty to verify this point? What is their ethical position?

It was concluded that discussions on these points had just begun, and no consensus had been reached.

## Is GOF research really safe?

### Initial problem raised

The issue of GOF research continued to smolder. On 20 December 2013, 56 scientists from more than a dozen countries sent a letter to European Commission President José Manuel Barroso calling for the initiation of a risk-benefit analysis of GOF research (Connor, 2013). The letter specifically asked for a scientific perspective on the significance of GOF research and included the following statement:

Fourth, there is little evidence for the claim that gain-of-function research can provide “critical information for the proper evaluation of candidate drugs.” Our 25 years of experience with HIV-1, another virus with a high propensity to mutate, has taught us that the only way to evaluate the efficacy of candidate antiviral drugs for RNA viruses is to conduct clinical trials. If ever H5N1 influenza went pandemic, we could only hope that the strain would be sensitive to some of the existing anti-influenza drugs. It would take several years to evaluate and get a new antiviral drug to market.

Thus, the necessity of developing a biosafety and biosecurity management system in GOF research was clarified. At the same time, there were calls for developing a governance system to manage the progress of safe research. However, Dr. Fouchier in the Netherlands and other groups conducting practical GOF research argued that academic freedom is hindered by the fact that the handling of experimental data to be published in papers is considered by the Dutch government to be the subject of export restrictions under trade control.

Meanwhile, other issues related to biorisk management arose. One report in June 2014 showed that approximately 75 employees at the Centers for Disease Control (CDC) in Atlanta might have been exposed to live anthrax due to inadequate safety measures and were consequently subjected to antibiotic treatment or health observation. The anthrax bacteria used in the biosafety level 3 (BSL3) laboratory were insufficiently inactivated, and samples supplied to other low-level BSL laboratories created the possibility of exposure to the researchers (CDC, 2014a). This case brought by experts from the CDC, the headquarters of infectious diseases research, had a huge social impact. In another case dating back to March of the same year, a low-pathogenic avian influenza H9N2 shipped to the Southeast Poultry Research Laboratories (SEPRL) of the United States Department of Agriculture was contaminated with a highly pathogenic avian influenza H5N1 that required handling at BSL3 (CDC, 2014b).

Another report detailed the July 2014 discovery of six freeze-dried vials of the variola virus in a Food and Drug Administration (FDA) laboratory at the NIH (FDA, 2016). Measures were immediately taken to transfer the vials to the CDC for further analysis and disposal under the supervision of the WHO. The question, however, was why those vials had remained—unnoticed—all those years in the FDA laboratory, even though it is a well-known fact that only the United States CDC and the Russian Vector are officially allowed to possess them. In response to the biosafety and biosecurity incidents occurring during that period, the US Government responded by implementing specific measures to optimize biosafety and biosecurity (OSTP, 2017; HHS, 2021).

The scope of these biosecurity measures was not limited to the management of pathogens but also extended to the risk assessment of the nature of the research itself, including GOF research. In this context, the United States government decided that it needed to assess the potential risks and benefits of certain types of GOF research with influenza, SARS, and MERS viruses (ePPPs) to inform the development and adoption of a new United States government policy governing the funding and conduct of GOF research (White House, 17 October 2014). As part of this process, the United States government also provided for a moratorium on new and current funding for some GOF research projects until this policy could be put in place (Reardon, 2014). Moreover, as a basis for the decision to lift the research ban, they decided to wait for the recommendations of the NSABB and the National Research Council.

### Debates and governance

A workshop titled “Potential Risks and Benefits of Gain-of-Function Research” was held by the National Academy of Sciences (NAS) in December of the same year (December 15–16, 2014) (National Research Council and Institute of Medicine, 2015). The potential benefits of GOF research were discussed in terms of surveillance, detection, and prediction, including its contribution to the detection of antigenic mutations and analysis to bridge the gap between surveillance, its contribution to the advancement of basic science, and its potential benefit to vaccine development. However, it was also argued that we need to delve deeper into the question of whether the research is truly meaningful. However, in the discussion of risk, Dr. David Relman, who co-chaired a National Research Council Report on “Globalization, Biosecurity, and the Future of the Life Sciences” (Institute of Medicine and National Research Council, 2006), stated that it is necessary to determine exactly which aspects of GOF research are of concern and listed the following six important considerations for risk assessment (Table 4).

**TABLE 4:** Key considerations in risk assessment (by David Relman).

•	The properties of newly created strains, their consequences, and alternative approaches;
•	Science and technology (S&T) trends over time;
•	The global distribution of risks and benefits, their relative weights, and questions of justice;
•	The types of possible misuse, in particular, safety and security;
•	Moral and ethical responsibilities of scientists and issues of public trust; and
•	Risk assessment and mitigation

One of the important points Dr. Relman made was that we need to carefully examine which parts of GOF research are high-risk and the possible consequences of neglecting these parts. While there may indeed be disadvantages of not being able to conduct the experiment, it is likely that many of the things the study is trying to prove can be achieved by alternative means. If there are facts that can only be obtained by conducting high-risk GOF studies, that rationale should be presented. Although the standard research content regarding this issue is available (NIH, 2013), there are no specific criteria for reviewing how the associated risks can be avoided. A background factor of the problem was suggested to be that scientific capacity is being distributed around the world and that governance and oversight should be distributed accordingly. For example, the reverse genetics technique developed by Palese et al., in 1996 (Palese et al., 1996; Palese and Roizman, 1996) is now no longer the technical prerogative of a particular laboratory. It is practiced in numerous research facilities and companies. In this context, the likely trend is that the risk of misuse and abuse is increasing. Importantly, this problem of infectious disease research is not limited to one country but is instead an international issue. As an important message, Relman stressed the need for both intellectual freedom and responsibility in science.

“Scientists have an important commitment to intellectual freedom and responsibility in science—and the two go hand in hand. This should include support for democratic and deliberative processes of decision-making.”- David Relman

The points to be considered from the risk/benefit arguments presented throughout this discussion are organized as follows (Table 5; National Research Council and Institute of Medicine, 2015).

**TABLE 5:** What to consider in terms of risk/benefit arguments for GOF research.

General		
•	Necessity of distinguishing between natural and intentionally combined information on pathogen genomes (including the pros and cons of information disclosure)
•	We are only at the beginning stages of learning the significance of GOF research; much more study is needed
•	How the National Science Advisory Board for Biosecurity (NSABB) could structure a more capacious and robust risk/benefit analysis
•	Are there any experiments that should be prohibited?
Biosafety perspectives	
•	The possibility of Laboratory Acquired Infections (LAIs) (due to factors such as inadequate procedures, controls, and hardware, or human errors)
•	Existence of pathogens not classified as Select Agents (e.g., MERS at the time of discussion)
•	Laboratory management problems, especially in developing countries [lack of biosafety policies, procedures, and training, personal protective equipment (PPE), and experiment supervisors]
•	Necessity of fostering a culture of safety (especially the difficulty in educating senior scientists)
•	Lack of practical advice on how to implement biosafety
•	There is a huge difference in the power of the Institutional Biosafety Committee (IBC) among research institutions
•	Acknowledging that risk reduction efforts in laboratories are acceptable, but that even low accident rates may be unacceptable for those with potentially enormous public health consequences—see Lipsitch and Inglesby (2014) and Lipsitch and Galvani (2014) alternatives to GOF studies
•	Lack of data collection on biosafety (collection of information on advanced containment facilities, creation of best practice repositories, etc.)
Biosecurity perspectives	
•	Recently, the focus of GOF research has shifted to biosafety, but biosecurity has a different perspective on risk assessment
•	There is considerable uncertainty in the key parameters of risk assessment, and the assessment needs to be periodically reviewed and updated

Because there are three perspectives in risk assessment: optimistic, pessimistic, and pragmatic, values themselves enter into the risk assessment process. Therefore, Gregory Koblentz of George Mason University said that “…it was essential to surface these assumptions early in any risk assessment process.” Carol Linden of the United States Biomedical Advanced Research and Development Authority stressed that “…a measured approach that recognizes that zero risk is not achievable can provide layers of protection to address legitimate safety and security concerns while continuing to make scientific progress…” was an important aspect of risk assessment. Nicholas Evans of the University of Pennsylvania commented that “…any deliberative process going forward would need to recognize the possibility of incommensurate values regarding the bounds of public health value, the value of innovation, and the value of security.” Furthermore, the essential problem of this issue was that the focus on the usability of research results by third parties is on scientists who follow the rules. In contrast, policing individuals who do not follow the rules is impossible (quotations in the preceding paragraph are from National Research Council and Institute of Medicine, 2015).

### New development

While the issue of GOF research on bird flu was heating up, research on the modification of the SARS virus was also underway. In 2013, a coronavirus (SHC014) with a close relationship to SARS-CoV, which uses human angiotensin-converting enzyme II (ACE2) in the receptor-binding domain of spike proteins to enter cells, was isolated from the horseshoe bat (Ge et al., 2013). Then, in 2015, using reverse genetics, a chimeric virus expressing this SHC014 spike in the backbone of a mouse-adapted SARS-CoV was created, which was shown to efficiently replicate in primary human airway cells by efficiently utilizing the SARS receptor, ACE2 (Menachery et al., 2015). This result was taken as proof of the existence of a virus that could cause a reemergence of SARS and was featured in *Nature Review Microbiology*’s Research Highlights under the heading “The next SARS?” (Nunes-Alves, 2016). On the other hand, this creation of a virus in the laboratory that could cause a pandemic triggered a new debate about whether this research is worth the risk.

Peter Daszak of the EcoHealth Alliance, who was involved in these experiments and had been studying GOF research, argues that “it helps indicate which pathogens should be prioritized for study.” Ralph Baric from the University of North Carolina at Chapel Hill, an author of the study, agreed. He said, “Our study shows that this virus has already overcome important barriers, such as its ability to attach to human receptors and efficiently infect human airway cells.” Conversely, Richard Ebright at Rutgers University in Piscataway, New Jersey refuted the significance of the study, saying, “The only impact of this work is the creation, in a lab, of a new, non-natural risk…” along with a scholar like Wain-Hobson who has long criticized GOF studies (Butler, 2015).

After the White House announced the suspension of federal public funding for GOF research in October 2014, the NSABB engaged in a series of discussions. The NSABB had also been tasked with examining whether the resumption of GOF research is really justified. In addition to the NSABB, this deliberative process included actions by the National Academy of Sciences, the NIH, and Gryphon Scientific. The NIH, therefore, commissioned Gryphon Scientific to conduct a full risk-benefit analysis of the GOF study and convened a meeting in January 2016 based on the approximately 1,000 pages of analysis received in December 2015 (draft as of December 2015; final report completed in April 2016) (Gryphon Scientific, 2016). However, due to the Christmas holidays, they felt that it was not possible to immediately obtain comments from the researchers and the general public, as no one had been given enough time to interpret the results of this analysis. Therefore, they invited the public to provide feedback to the NSABB and participate in the National Academy’s forum on GOF research to be held in March 2016.

After much debate, review, and discussion (Casadevall and Shenk, 2014; Imperiale and Casadevall, 2014; Schultz-Cherry et al., 2014; Wain-Hobson, 2014; Duprex et al., 2015), the NSABB voted unanimously at its May 2016 meeting to finalize the working group’s draft recommendations with some minor edits. The final result was “A Report of the National Science Advisory Board for Biosecurity: recommendations for evaluating and oversight of proposed gain-of-function research” (May 2016) (NSABB, 2016). This report was the final decision on the nature of GOF research, a culmination of 5 years of discussion. The main points are as follows (Tables 6, 7).

**TABLE 6:** NSABB findings.

**Finding 1.** There are many types of GOF studies, and not all of them have the same level of risk. Only a small subset of GOF research—GOF research of concern (GOFROC)—entails risks that are potentially significant enough to warrant additional oversight
**Finding 2.** The U.S. government has several policies to identify and manage risks associated with life sciences research. There are several points throughout the research life cycle where, if the policies are implemented effectively, risks can be managed, and oversight of GOF research of concern could be implemented
**Finding 3.** Oversight policies vary in scope and applicability and do not cover all potential GOFROC; therefore, current oversight is insufficient for all GOF research of concern
**Finding 4.** An adaptive policy approach is a desirable way to ensure that oversight and risk mitigation measures remain commensurate with the risks associated with the research and that the benefits of the research are being fully realized
**Finding 5.** There are life sciences research studies, including possibly some GOF research of concern, that should not be conducted because the potential risks associated with the study are not justified by the potential benefits. Decisions about whether specific GOFROC should be permitted will entail assessing the potential risks and anticipated benefits associated with the individual experiment in question. The scientific merit of a study is a central consideration during the review of proposed studies. Still, other considerations, including legal, ethical, public health, and societal values, are also important and need to be taken into account
**Finding 6.** Managing risks associated with GOF research of concern, like all life sciences research, requires both federal and institutional oversight, awareness, compliance, and a commitment by all stakeholders to safety and security
**Finding 7.** Funding and conducting GOF research of concern encompasses many international issues (Excerpted from the NSABB’s report (2016). Emphasis was added by the authors)

**TABLE 7:** NSABB recommendations to the United States government.

**Recommendation 1.** Research proposals involving GOF research of concern entail significant potential risks and should receive an additional, multidisciplinary review prior to determining whether they are acceptable for funding. If funded, such projects should be subject to ongoing oversight at the federal and institutional levels
As part of this recommendation, the NSABB has proposed a conceptual approach for guiding funding decisions about GOFROC. First, the NSABB identified the attributes of GOFROC, which is research that could generate a pathogen that is: 1) highly transmissible and likely capable of wide and uncontrollable spread in human populations; and 2) highly virulent and likely to cause significant morbidity and/or mortality in humans. Next, the NSABB identified a set of principles that should guide funding decisions for GOFROC. Only research determined to be in line with these principles should be funded. Additional risk mitigation measures may be required for certain research studies to be deemed acceptable for funding
**Recommendation 2.** An advisory body designed for transparency and public engagement should be utilized as a part of the U.S. government’s ongoing evaluation of oversight policies for GOF research of concern
**Recommendation 3.** The U.S. government should pursue an adaptive policy approach to help ensure that oversight remains commensurate with the risks associated with the GOF research of concern
**Recommendation 3.1.** The U.S. government should develop a system to collect and analyze data about laboratory safety incidents, near-misses, and security breaches, as well as the effectiveness of mitigation measures to inform GOF research of concern policy development over time
**Recommendation 3.2.** The U.S. government should develop or facilitate the development of a system to collect and analyze data about Institutional Review Entity (IRE) challenges, decisions, and lessons learned to provide feedback to the IRE community and inform policy development for GOF research of concern over time
**Recommendation 4.** In general, oversight mechanisms for GOF research of concern should be incorporated into existing policy frameworks when possible
**Recommendation 5.** The U.S. government should consider ways to ensure that all GOF research of concern conducted within the U.S. or by U.S. companies is subject to oversight, regardless of funding source
**Recommendation 6.** The U.S. government should undertake broad efforts to strengthen laboratory biosafety and biosecurity and, as part of these efforts, seek to raise awareness about the specific issues associated with GOF research of concern
**Recommendation 7.** The U.S. government should engage the international community in a dialogue about the oversight and responsible conduct of GOF research of concern
(Excerpted from the NSABB’s report (2016). Emphasis was added by the authors)

In response to the NSABB’s recommendations, staff from the White House Office of Science and Technology Policy (OSTP) and the National Security Council took the lead in an interagency effort to develop a policy that would permit the lifing of the 2014 moratorium. This policy was released in January 2017, when OSTP issued “Recommended Policy Guidance for Departmental Development of Review Mechanisms for Potential Pandemic Pathogen Care and Oversight (P3CO)” (https://www.phe.gov/s3/dualuse/documents/p3co-finalguidancestatement.pdf). The OSTP P3CO Guidance set out a procedure for reviewing proposals to conduct ePPP research, and it specified that if a U.S. government department or agency established a review process consistent with that procedure, it would be able to resume ePPP research. The procedure specified by OSTP largely followed that recommended by the NSABB. Finally, in December of 2017, the United States NIH issued a “Framework for Guiding Funding Decisions about Proposed Research Involving Enhanced Potential Pandemic Pathogens” (HHS, 2017), which satisfied the OSTP guidelines and lifted its funding suspension for GOF experiments on influenza, SARS, and MERS viruses which had been in place since October 2014 (Collins, 2017).

This paper focuses on the U.S. policy-making process regarding DURC and GOF because very few such processes exist in other countries. As the 2021 Global Health Security Index observed, “94% of countries have no national-level oversight measures for dual-use research, which includes national laws or regulation on oversight, an agency responsible for the oversight, or evidence of a national assessment of dual-use research” according to the 2021 Global Health Security Index (Bell and Nuzzo, 2021). Further, the state of governance in countries other than the United States and the roles of international organizations such as WHO, WOAH (World Organisation for Animal Health), and Biological Weapons Convention (BWC) in the future governance of infectious disease research are quite important to build a safe and secure society in terms of life science research and its dual-use concerns.

## What we learned about infectious disease research from our experience with COVID-19

On 7 November 2005, the InterAcademy Panel (IAP) issued its biosecurity recommendations, quoting the words of F. Rabelais “Knowledge without conscience is simply the ruin of the soul (*Science sans conscience n’est que ruine de l’âme*. F. Rabelais, 1532)”, and stated that “…scientists should refuse to undertake research that has only harmful consequences for humankind.” This IAP statement was issued in anticipation of the Conference of the States Parties to the Biological Weapons Convention held in Geneva in December of the same year. It was endorsed by the science academies of more than 60 countries. Scientists have a special responsibility with regard to the dual use of science and technology. Table 8 presents five principles from the IAP Statement on Biosecurity (IAP, 2005).

**TABLE 8:** Five principles of the IAP Statement on Biosecurity.

1	**Awareness.** Scientists have an obligation to do no harm. They should always take into consideration the reasonably foreseeable consequences of their own activities. They should, therefore:
	a) Always bear in mind the potential consequences—possibly harmful—of their research and recognize that individual good conscience does not justify ignoring the possible misuse of their scientific endeavor;
	b) Refuse to undertake research that has only harmful consequences for humankind
2	**Safety and Security.** Scientists working with agents such as pathogenic organisms or dangerous toxins have a responsibility to use good, safe, and secure laboratory procedures, whether codified by law or common practice
3	**Education and Information.** Scientists should be aware of, disseminate information about and teach national and international laws and regulations, as well as policies and principles aimed at preventing the misuse of biological research
4	**Accountability.** Scientists who become aware of activities that violate the Biological and Toxin Weapons Convention or international customary law should raise their concerns with appropriate people, authorities, and agencies
5	**Oversight.** Scientists responsible for research oversight or evaluation of projects or publications should promote adherence to these principles by those under their control, supervision, or evaluation and act as role models

In the December 2017 decision (Collins, 2017) to lift the freeze on funding for GOF research, the NIH’s Director Francis S. Collins stated:

“We have a responsibility to ensure that research with infectious agents is conducted responsibly and that we consider the potential biosafety and biosecurity risks associated with such research. I am confident that the thoughtful review process laid out by the HHS P3CO* Framework will help facilitate the safe, secure, and responsible conduct of this type of research in a manner that maximizes the benefits to public health.”

-*P3CO: Potential Pandemic Pathogen Care and Oversight

### Useful measures

In light of these statements, now that we are in the midst of the COVID-19 pandemic, it is time for us to properly examine the real advantages and disadvantages of GOF research. Between the lifting of the freeze on GOF research funding in 2017 and the declaration of the COVID-19 pandemic by WHO on 11 March 2020, how has GOF research using the SARS coronavirus been conducted, and what have been the results? Because of the short period during which these GOF experiments have been conducted, it may not be appropriate to make general statements about the work results. Still, at least there is no evidence that the study was effective in predicting epidemic strains or selecting vaccine strains. As the moratorium was not conducted on a global scale, there may have been a number of other forms of GOF studies conducted under the surface during this period. In addition, the virus examined by the EcoHealth Alliance study (Dance, 2021), which has been discussed in some circles, was very different from the currently prevalent SARS-CoV-2, and it is unlikely that this was the direct cause of the outbreak. However, even with these considerations, the GOF study has not shown that it is effective in dealing with the pandemic in a way that significantly outweighs the risks. Of course, we do not deny the importance of scientific inquiry, and we are aware that truly meaningful research results can only be produced through broad-based research activities. However, at present, we do not even know the true origin of SARS-CoV-2, the pathogen of COVID-19, including whether the pandemic may have been a consequence of a laboratory accident involving SARS-like coronaviruses that were being studied, or even genetically engineered, in the laboratory or through which host it was transmitted to humans.

Considering the current disastrous state of COVID-19, it is clear that the technologies necessary for early diagnosis, the mechanisms for accurately and rapidly grasping the epidemic and mutation status, the prediction of the spread of infection through epidemiological analysis and mathematical modeling, the development of technologies for therapeutic agents and treatments, and the public health measures and vaccine development necessary for the prevention, are of primary importance. Technologies and related research to produce countermeasures against COVID-19 (shown in Table 9) have contributed greatly to these efforts.

**TABLE 9:** Items required for COVID-19 countermeasures and supporting technology and research.

Countermeasures against COVID-19	Technology and research areas
Early diagnosis	Identification of genomic information, RT-PCR, Rapid test for antigens
Establishment of diagnostic methods	Case Collection, Analysis of clinical laboratory data, Pulse oximeter, Detection of pneumonia image by CT
Monitoring the epidemic situation	Report from hospitals, Systems of public health authorities such as health centers, Compilation of a database
Understanding mutations	Viral genome sequencing, Mutation-specific PCR, Measurement of neutralizing antibodies
Epidemiological analysis	Viral genome sequencing, Centralized management, Disclosure of information (such as GISAID and GenBank), Case reporting system
Epidemic forecast	Mathematical models, Use of digital devices (such as measuring human flow with mobile phones)
Therapeutic drugs	Genome-based drug discovery, Drug repositioning, Antibody Drugs, Symptomatic therapy (steroids, anticoagulant therapy)
Treatment method	Oxygen inhalation, Ventilator management, Extracorporeal membrane oxygenation (ECMO)
Public health countermeasures	Urban lockdowns, Patient Isolation, Remote working, Avoidance of the 3C’s (closed spaces, crowded places, close-contact settings), Wearing a mask, Disinfection with alcohol, and Personal protective equipment (PPE) for health care providers, etc.
Vaccine development	Vaccine platform development, mRNA vaccine, Recombinant virus vector vaccine, Recombinant protein vaccines, Traditional methods (inactivated vaccine, live attenuated vaccine)
Crisis management/Outreach	Hospital management, Risk communication, Crisis communication

The most promising medical countermeasure against the current COVID-19 pandemic is the development of vaccines. Particularly striking at present was the development of the mRNA vaccine (Kariko et al., 2005), introduced as a new technology, that enabled mass production of an approved vaccine in less than 1 year from the discovery of the pathogen it countered. This technology is the result of decades of research, extending long before COVID-19. The availability of genomic information and the sequencing of the most efficient target portion of the genome―in the case of SARS-CoV-2, its spike protein―enabled vaccine design. Another useful technology is a recombinant adenovirus vector vaccine, which was also used during the successful development of a vaccine against the Ebola virus (Zhu et al., 2017). The fact that these vaccines were made available for practical use, including in clinical research, less than a year after the COVID-19 outbreak is due to the fact that the process from vaccine design to actual formulation does not require much time, as long as the genomic information of the virus is available. Indeed, the bulk of that year-long interval comprised preclinical and clinical testing; the actual development of the RNA sequence for the vaccine was done within days of obtaining the SARS-CoV-2 genome sequence. Another advantage is that even if a mutant virus appears, only the sequence design for the vaccine needs to be changed so that the second generation vaccine can be prepared quickly. These novel technologies have a significant advantage in terms of time savings compared to the generation of conventional inactivated or attenuated live vaccines. This novel type of vaccine has been proven to be highly effective with relatively few side effects, as shown by the vaccination results (Polack et al., 2020; Sahin et al., 2020; Arbel et al., 2021).

We can now retrospectively determine the importance of early diagnosis of patients, isolation and identification of pathogens, and containment of local infections as of November 2019, before the spread of COVID-19. In contrast, the GOF work, as well as all other SARS research results combined, did not provide the scientific basis for these actions to be performed rapidly—perhaps because there was no direct GOF research on SARS-CoV-2 and SARS virus is different from SARS-CoV-2.

However, next-generation sequencing was very powerful in the genome analysis of SARS-CoV-2. In particular, next-generation sequencing contributed to the identification of virus isolates from patients with pneumonia of unknown origin, the early establishment of RT-PCR diagnostic methods, and the accumulation of sequencing data, mainly from the Global Initiative on Sharing All Influenza Data (GISAID - hCov19 Variants) and National Center for Biotechnology Information (NCBI) in the United States, which was used effectively as a tool to visualize changes in the viral genome and to accurately determine the prevalence and time course of mutant strains. Next-generation sequencing has the advantage of being able to monitor the situation of epidemics in any country, which can help to change our public health measures accordingly by comparing our situation with the scenario in different countries.

### Less useful/not approved effective measures

In addition to this, a basic analysis of the nature of new coronavirus mutations would have helped us understand how mutations change virulence. For example, early in the pandemic, SARS-CoV-2 acquired a mutation called D614G, which conferred slightly greater infectivity to the virus than the original. The D614G spike mutation increased the efficiency of intracellular entry by increasing ACE2 binding affinity (Ozono et al., 2021). The D614G mutation contributes to the adaptation of the virus for increased multiplication and high transmission of infection between animals (Hou et al., 2020).

The epidemiological background inferred fromexplosive epidemic of COVID-19 may be that viral evolution leads to greater infectivity and contagiousness or evasion of protective immunity against infection over time; genomic analysis has revealed the nature of the virus in this regard (Table 10) (Rettner, 2022). For example, the L452R mutation is characteristic of the delta mutant strain that raged in India during April and May of 2021 and then spread to other parts of the world. This mutation of the 452nd amino acid of the spike protein, which binds to the ACE2 receptor when the virus enters the cell, from L (leucine) to R (arginine), triggers massive expansion of SARS-CoV-2 variants (Tchesnokova et al., 2021) and also increases infectivity by helping the virus evade the host immune system (Motozono et al., 2021). The study that predicted over 100 mutations and analyzed their impact on infectivity and antigenicity (Li et al., 2020) showed that L452R resulted in weak binding to neutralizing antibodies. They generated vesicular stomatitis virus (VSV)-based pseudotyped viruses using site-directed mutagenesis to create various mutations on the receptor-binding domain and tested their sensitivity to convalescent serum samples as well as infectivity in cultured cells. Some of these, such as D614G and L452R, were involved in actual epidemics, whereas others, such as N501Y and E484K, were not necessarily predicted to be mutant strains that caused major epidemics. As they did not use actual SARS-CoV, the technique used in their study was considered an alternative method for GOF/ePPP research.

**TABLE 10:** Coronavirus variants identified during the COVID-19 outbreak.

Variant name by WHO	Country first identified	Lineage name	Variant of concern (VOC)/Variant of interest (VOI)	Type of mutation
Alpha variant (α)	United Kingdom	B.1.1.7	VOC	N501Y
Beta variant (β)	South Africa	B.1.351	VOC	N501Y, E484K
Gamma variant (γ)	Brazil	P.1	VOC	N501Y, E484K, 10 amino acid changes in the spike protein
Delta variant (δ)	India	B.1.617.2	VOC	L452R
Omicron variant (o)	South Africa	B.1.1.529	VOC	More than 30 mutations in the genes that code for its spike protein, with ten of those genes coding for parts of the “receptor-binding domain,” or the part of the spike protein that latches onto human cells
Lambda variant (λ)	Peru	C.37	VOI	G75V, T76I, del247/253, L452Q, F490S, D614G and T859N
Mu variant (μ)	Columbia	B.1.621	VOI	E484K, K417N

According to Live Science (8 June, 2022).

### GOF (mainly not immediate useful)

One of the beneficial objectives of the risk-benefit analysis of GOF studies was that the virus produced could be used to predict vaccine strain selection. However, with the advent of the new technology of “viral genome sequencing → identification of target sites → mRNA vaccine creation,” it became clear that the process of producing human-infectious viruses in GOF experiments and selecting vaccine strains from among them was not a useful technique for developing vaccines quickly, efficiently, or practically.

In the development of new drugs, it is necessary to use actual viruses in the screening of drug effects, analysis of drug mechanisms, and testing for side effects. In contrast, in GOF research, some believe that the viruses created can be used for drug development in advance of an outbreak. However, before a drug can actually be administered to humans, it is necessary to identify the prevalent strains, confirm drug efficacy against them, and conduct clinical studies to determine whether the drug is actually effective and also whether it causes any side effects. During this course, the most time-consuming part is the clinical research stage, and we must wait for the results of this clinical research to ensure that the drug can be used as a therapeutic drug for many people. Therefore, the purpose of GOF research would be to predict the infectivity of the virus in humans and to precautionarily investigate what drugs could be used as therapeutic agents against the virus. Still, it is necessary to take into account that this research is only a preliminary step to practical use. In addition, even if a GOF study shows that a drug is effective against the virus it was created for, there is no guarantee that it will be effective against the virus that actually causes the epidemic. This is especially true for antibody drugs, for which small changes in the viral protein sequence can have significant consequences in terms of antibody binding.

In summary, the most outstanding achievement of the GOF study of the SARS virus was showing that coronaviruses from horseshoe bats other than SARS-CoV can infect humans. However, such a hypothesis is just within the predictable range based on the binding of human ACE2 to the viral spike protein, and it shows that the GOF study has not added any new meaning. In addition, the selection of vaccine strains and the prediction of epidemic strains have not been achieved, leaving unanswered the question of whether this pandemic provides justification for artificially creating a PPP that does not exist in nature.

## Future governance of infectious disease research

As we described earlier, we sympathize with the idea that “research that can only cause harm and cannot be proven beneficial should not be conducted,” as mentioned by the IAP in its biosecurity recommendations. However, the seven research categories in the Fink report are indispensable when discussing how to conduct pathogen research. In other words, while it is undoubtedly true that these are high-risk research methods in terms of dual-use, the problem cannot be solved by uniformly banning all of them. Accordingly, it is conceivable that these tools could help investigate the nature of pathogens and the essential etiology of infectious diseases, so it is necessary to consider the disadvantages of not conducting research using these tools to gain scientific knowledge. This creates an inherent dilemma in that the dual-use problem cannot be solved straightforwardly. Considering the state of GOF research, we must once again examine what conditions are required to render GOF research necessary from the three potentially different perspectives of genuine academic truth-seeking, social demands, and practical applicability and usability. The current COVID-19 pandemic seems to have provided the perfect opportunity to reconfirm these conditions.

Here what is once again required of GOF research is the disclosure of information and transparency regarding the status of research implementation and various issues that have emerged in the course of the research. An institutional governance structure is necessary to support GOF research. If we are to promote research that is safe and contributes to public health, the risks and concerns should be identified in advance for each experimental item, and countermeasures should be clearly stated before proceeding. A system should be created that can respond quickly to new problems that arise during the course of the experiment. The problem comes when researchers withhold or hide information when an inconvenient situation arises. Individual researchers should raise their awareness of the dual-use issue and truly understand and practice appropriate biosecurity codes of conduct. However, a system should also be established across the entire research facility with the aim of promoting safe and fair research. As such, responsible research is seen as an obligation that each researcher owes to his or her colleagues, all of whom will bear consequences if any one of them is responsible for a high-consequence incident rather than having only individual researchers punished or strongly criticized whenever an inconvenient event occurs.

Therefore, when these ideas are incorporated at the specific individual research level, the role of the institutional ethics committee in each research facility is to specify what criteria should be used to approve GOF research. In particular, a clear explanation of the benefits and risk avoidance in the risk-benefit analysis is required. For the content of the benefits, in addition to the opinion of the applicant researchers, supplementary opinions of researchers in related fields of expertise are important. Risk concerns should not only be addressed from the perspective of experts, but also the opinions of the general public should be incorporated with respect to these concerns.

In response to the situation during the COVID-19 pandemic and the need for an effective global response to biological threats, a meeting of G7 experts (G7 Experts’ Meeting on Strengthening Laboratory Biorisk Management) was held in October of 2020. A comprehensive discussion was held on infectious disease research, including biosafety and biorisk management in advanced containment research facilities. The meeting concluded with 11 recommendations for two issues: “Advance the evidence base of laboratory biorisk management practices and procedures” and “Advance transparency about laboratory biorisk management practices and procedures” (Table 11). This may not be directly related to the specific GOF study producing the ePPP, but it is still important as it promotes strict pathogen control.

**TABLE 11:** Recommendations by the G7 experts’ meeting on strengthening laboratory biorisk management.

**Issue 1:**	**Advance the evidence base of laboratory biorisk management practices and procedures**
1	International experts’ workshop(s) to develop a research agenda
2	Ongoing international working group on evidence-based laboratory biorisk management
3	Research agenda projects to be addressed in Global Partnership projects
4	Groups to sponsor forums on evidence-based laboratory biorisk management
5	Research groups to incorporate applied biorisk research topics
**Issue 2:**	**Advance transparency about laboratory biorisk management practices and procedures**
6	Laboratories to follow and/or harmonize with international guidelines for biosafety procedures
7	Confidence Building Measures of the Biological Weapons Convention to include BSL4 laboratory oversight
8	Containment labs to exchange best practices and lessons learned
9	Exchange lessons learned, plans, and appropriate information for laboratory biorisk management
10	Relevant groups to develop and disseminate best practices
11	Share experiences in biorisk management training among universities, and professional groups

## Conclusion

As can be inferred from the above analyses, the key to effective and safe public health research on infectious diseases is to provide opportunities to discuss what the problems are, to take a clear stance on the nature and safety of research and research ethics, and to disseminate and share this information. In addition to these ideas, we must not forget the importance of the continued involvement of all stakeholders, especially experts who are well versed in infectious disease research. From this perspective, GOF research is recognized as a priority issue of the kind that deserves re-examination and discussion involving many stakeholders at the institutional, national, and international levels.

Public trust in science is a critical component of continued scientific research, which requires transparency, communication, outreach, surveillance of laboratory-derived infectious diseases, public health partnerships, and institutionalized ethics (Kahn, 2021). In reviewing GOF research, the importance of adequately examining the research items that enabled a rapid response to the pandemic is indisputable. As far as our current environment is concerned, the acquisition of viral genome information, its application to vaccine strategies, and rapid public health measures were the most important factors in resolving the spread of infection, which comprise a very different approach from that of GOF research. Thus, it clearly suggests that GOF is ineffective in dealing with the pandemic, at least in the early stage and rapid responding to COVID-19 but may have great potential for misuse. Therefore, the risks of GOF research are clearer now than when it was debated earlier, whereas the pandemic has shown that its benefits may be less significant. Therefore, it is now time to rethink the risk/benefit calculus of GOF research.

Life science technology in the field of infectious disease research is constantly evolving. The issues that need to be addressed and the demands of society are also subject to change, depending on the situation. A decision at any one time is not always the right one for the future, and certain regulations and rules are not applicable in all cases. Still, it is imperative that the governance for GOF research on ePPP is based on this understanding and that it be deliberated on continuously. Therefore, GOF research needs to be revisited and assessed for its effectiveness in dealing with potentially pandemic infectious diseases in the future as more information becomes available.
